# Supplementary material for: A highly reactive precursor in the iron sulfide system
Source: Nat Commun. 2018 Aug 7;9:3125. doi: 10.1038/s41467-018-05493-x (PMC6081449; doi:10.1038/s41467-018-05493-x)
Supplement: Supplementary file 1 — Supplementary Information [file 41467_2018_5493_MOESM1_ESM.pdf]

Supplementary Information

A Highly Reactive Precursor in the Iron Sulfide System

Matamoros-Veloza *et al.*

Supplementary Methods

Preparation of O2-free water

O_2-free water was prepared freshly for each experiment using 18.2 MΩ.cm Milli-Q water from a Milli-Q Academic system. The water was boiled under continuous stirring in specially designed scrubber flasks, while bubbling continuously with O_2-free N_2 gas (grade 99,9995%). To produce 1L of water, we boiled it for 2 hours and then left it to cool down for another 2 hours, while continuously bubbling with the O_2-free N_2 gas. Once cooled to room temperature, the scrubber flask was sealed and the deoxygenated water was immediately transferred into the vacuum interlock of the glove box (CoyLaboratory Products Inc.). There, 6 cycles of vacuum/N_2 injection were applied to remove any oxygen, prior to insertion into the anaerobic chamber, in which the anaerobic environment was maintained with a N_2/H_2 mix (95:5 %) at a H_2 concentration of 3.8%. The O_2 free atmosphere was kept in the chamber through the standard Coy Lab catalyst, made of Al_2O_3 pellets coated with palladium. Any oxygen present in the chamber, readily reacted with the H_2 in the glove box to form water that adsorbs onto dried silica gel.

Inside the anaerobic chamber, an anaerobic gas monitor unit was placed, equipped with a sound alarm when O_2 concentrations raised above 1 ppm. Throughout our experimental work, this monitor never showed any sign of oxygen in the chamber. In addition, prior to using the as above prepared deoxygenated water in an experiment, we tested an aliquot by analyzing its dissolved oxygen concentrations using a Hack HQ30D portable DO meter and a new factory calibrated luminescence / optical DO probe.

Supplementary Discussion

XRD results

In order to obtain information about the crystallite sizes, the peak at ~12 Å (~ 7 ˚ 2θ) from a titration experiment shown in the main text in Fig. 1a was fitted using a Pseudo-Voigt profile (Supplementary Fig. 4a). The best fit was obtained with two peaks with integral breadths 0.3614 and 0.2071 yielding crystallite sizes of ~ 23 and ~44 nm (Supplementary Fig. 4a). These calculated sizes are far larger than the ~2 nm nanocrystal sizes observed in TEM. These bigger crystallite

sizes derived from the bulk XRD likely reflect the fact that the individual nanocrystals seen in TEM self-assembled and fused together without phase transformation during XRD sample preparation and handling; this fact was also occasionally observed in our TEM images (see for example Supplementary Figure 4b and c).

Lattice spacings of 12.3 Å and 12.1 Å were calculated from the fits for the two crystallite sizes suggesting that both have a layered structure. Similar *d*-spacings were derived from the FFT of the TEM images of the individual particles confirming that the same phase was imaged. The XRD patterns however did not revealed a distinctive and singular peak with an integral breadth that could be interpreted as characteristic for 2 nm particles. This could be because the signal to noise ratio of such particles would likely be so weak but also because self-assembly and agglomeration of particles during sample preparation and drying for the XRD analysis could have caused aggregation. Furthermore, during the TEM analysis we targeted specifically the small size fraction and imaged primarily thin areas of a sample characterized by an abundance of smaller and individual particles.

XRD data from a diffusion experiment (Fig. 1a in main text, red pattern) agrees to a large extent with the XRD data from the titration experiment (Supplementary Fig. 4a). Moreover, the hump at ~8.2 Å in the XRD pattern of the diffusion experiment also supports the notion of a larger sized and poorly crystalline phase likely also the consequence of the evolution from the FeS_{nano} to an aggregated short range ordered phase. In both cases the XRD-derived crystallite sizes likely indicate the sample-handling induced aggregation of the precursor phase (FeS_{nano}) to a phases characterized by larger crystallites sizes. However and importantly, our data (Fig. 1a main text) also clearly reveals that the XRD patterns were different from mackinawite (lack of the 5.0 Å peak).

Raman results

The Raman spectra of FeS_{nano} (Fig. 2a and Supplementary Fig.5) showed two broad, but weak bands at 204 and 215 cm^{-1}, assignable to Fe-S asymmetric stretching vibrations from monosulfides (Fig. 2a; Supplementary Table 4). The broadening of the deconvoluted peak at 204 cm^{-1} indicates a high level of disorder at the local bond scale and a long range disorder. This peak is redshifted

relative to the Fe-S asymmetric vibration in mackinawite (Supplementary Fig. 6; Supplementary Table 5), reflecting a weakening in the interionic bonding caused by an expansion in the lattices (1), supporting our XRD and TEM evidence (Fig. 1; Supplementary Table 3). The additional Fe-S band at 274 cm^{-1} reflects the symmetric Fe-S stretching vibration (Fig. 2a; Supplementary Table 4) in our new FeS_{nano} phase. This symmetric stretching vibration band appears very intense and sharper in mackinawite at 272 cm^{-1} (Supplementary Fig. 6), while the asymmetric Fe-S stretching vibration band is centered on 210 cm^{-1} (Supplementary Fig. 6) (2, 3). These two vibrations are different to the Fe-S bands characterizing our new FeS_{nano} phase (Fig. 2a and Supplementary Fig.5; Supplementary Tables 4, 5).

XPS results

Initially, the XPS spectra of the FeS_{nano} phase were fitted only with the minimum peaks possible (i.e., using only one doublet for a monosulfide species). However, the quality of these fits was very poor and improved significantly when using two doublets for monosulfides (62%, Supplementary Table 6), and one doublet each for disulfides (14%) and polysulfides (17%). The monosulfide species were identified through their binding energies (i.e., 161.1 and 161.8eV; Supplementary Table 6). The XPS data indicated that sulfur atoms in FeS_{nano} were present in two slightly different chemical environments that could reflect the presence of different atomic coordination arrangements (4). Furthermore, two different monosulfide chemical environments were also identified in the mackinawite spectra with Fe^{II}-S and Fe^{III} species present (Supplementary Table 6).

It was not possible to calculate the Fe^{II}:S ratios from the XPS spectra due to the large excess of sulfur present, which yielded unreasonably high values. The excess sulfur could be a result of either the presence of excessive polysulfides or indicate a fully saturated sulfur layer on the surface of the analyzed samples. In addition, higher S/metal ratios (i.e., 3.2) estimated by XPS have been previously documented in *in-situ* fractured pyrite, and attributed to the presence of polysulfides on the pyrite surface (5).

It is important to highlight again that in none of the XPS spectra collected from the FeS_{nano} or mackinawite did we observe signs of air oxidation (lack of any signal in the O spectra; see Fig. 2d

and Supplementary Fig. 7c). This confirms that indeed all our extreme and careful measures to maintain anaerobicity throughout all experimental, sample handling and sample transfer procedures into the XPS ensured the preservation of the needed highly reduced environment.

Combining Raman with X-ray photoelectron spectroscopic (XPS) analyses of FeS_{nano} also revealed the presence of variable proportions of long and short chain polysulfides (e.g., S_2^{2-}, S_3^{2-}, S_4^{2-}, S_n^{2}; Figs 2a and b) in its structure. Raman vibrations at 148, 170 and 553 cm^{-1}, characteristic of disulfides, were matched by polysulfide vibrations at 162, 228, 323, 378 and 463 cm^{-1} (Fig. 2a; Supplementary Table 4). The proportions of di- (S_2^{2-}) and polysulfides (S_n^{2-}) in the FeS_{nano} phase contributed 14 and 17 % respectively to the signal calculated from the integrated intensity, while the largest contribution (up to 62 %) was from monosulfide species (S^{2-}) (Fig. 2b; Supplementary Table 6).

Invariably the presence of polysulfides and Fe^{III} species in FeS_{nano} raise the question whether are the result of air oxidation during sample handling. However, our data show no Fe-O or S-O species in either Raman or XPS spectra supporting our strict anaerobic procedures throughout sample preparation, handling and analyses (Fig. 2d, Supplementary Fig. 5 and 6). We tested for signs of change by etching the sample surface with argon. The post etching XPS spectra demonstrate that the species present in the solids remained unchanged; however, the proportion of Fe and S species revealed changes less than 1 % (Supplementary Table 6). For example, the Fe^{II} surface composition decreased from 58 to 57%, while Fe^{III} increased from 42 to 43%; moreover, the contribution of monosulfides increased from 67.8 to 68.2%, of polysulfides from 18.1 to 18.7%, while disulfides decreased from 14.9% to 13.0%. These changes are minor indicating that the species and contributions of Fe^{II}, Fe^{III}, as well as the monosulfides, disulfides and polysulfides are not an artifact but that they are an integral part of the structure of the FeS_{nano} phase. Just to mention again, the O1s spectrum showed a total lack of Fe-O or S-O species confirming full anaerobicity during the XPS analyses.

The presence of Fe^{III} has been previously documented in mackinawite ($Fe^{II}_{1-3x}Fe^{III}_{2x}S$) (2, 6), and Fe^{III}-containing mackinawite was shown to form under acidic conditions (pH 3.5) when reacting low pH Fe^{2+}aq and high pH S(-II) solution (6, 7). At these conditions (nominal pH ~3.5), oxidation of mackinawite occurs by H_2S via dissolution/re-precipitation mechanism (6). In this

mechanism any formed mackinawite dissolves rapidly causing the oxidation of Fe^{II} to Fe^{III} in the structure, and triggers the releasing of Fe^{II} into solution to balance the mackinawite structure.

Density functional theory (DFT) calculations

In order to understand the interactions at play between the FeS_{nano} layers and the polysulfides of the iron sulfide precursor, we have investigated by means of density functional theory (DFT) the intercalation of H_2S_2 and H_2S_4 into mackinawite. Both S_2^{2-} and S_4^{2-} species have been detected experimentally, while the hydrogen atoms were added to enforce charge neutrality in the simulation cell. Being formed by FeS_4 tetrahedra and thanks to its layered structure, mackinawite is the most suitable candidate among the iron sulfides for modelling the intercalation of guest species. We have generated a number of input geometries by inserting the gas-phase optimized molecules in a 3x3x1 mackinawite supercell built by replicating the DFT relaxed unit cell in reference (25) and expanding the c lattice constant to 8.0 Å. We have also included trial structures with one S atom of the intercalated species at covalent bond distance from the atoms of the FeS_4 tetrahedra. All optimizations were performed with VASP 5.3 (26, 27), taking into account the Van der Waals correction as implemented in the DFT-D2 method of Grimme (28). As in previous works on mackinawite (25, 29), all the calculations were non spin-polarized. We employed the PBE functional (30), together with the projector augmented wave method to model the core-electron interaction (31), treating explicitly the 4s, 3d and 3p electrons of Fe, the 3s and 3p of S, and the 1s of H. All the optimizations were performed with a plane wave cutoff of 500 eV and a 2x2x3 Monkhorst-Pack grid (32), and stopped when the force acting on each ion was less than 0.03 eV/Å. Density of states were calculated with a mesh of 4x4x6.

The most energetically stable configurations obtained by relaxing the ions, as well as the volume and shape of the simulation supercells (Supplementary Fig. 8a,b) show that the loss of the initial tetragonal symmetry provides the guest molecules with newly formed intercalation cavities. This resulted in an interlayer expansion that increases with the length of the polysulfide chains with the *c* lattice constants reaching 8.0 and 8.7 Å for H_2S_2 and H_2S_4 intercalations respectively. All the S-S distances are comparable or larger than 3.6 Å (i.e., twice the Van der Waals radius of S) (33) suggesting that trapped polysulfides only interacts with the Fe-S layers via weak dispersion forces (Supplementary Fig. 8c) (34). The DFT results demonstrated that energetically the presence

of polysulfides intercalating Fe-S tetrahedral sheets as observed in the FeS_{nano} is a possible configuration to explain our observations.

XANES results

The full Fe K-edge XANES spectra from FeS_{nano} and mackinawite showed three distinguishable features in the regions marked as (I), (II) and (III) corresponding to a pre-edge (1s-3d), an increase in the energy of the edge jump (1s-4s) and the near-edge (1s-4p) (Fig. 3 and Supplementary Fig. 9). At a first glance, the overall fingerprint of the FeS_{nano} spectrum is different to that of mackinawite. The pre-edge is less pronounced and there is a clear difference in the shape of the main peak of the spectra. The mackinawite spectrum from our sample is in most features very similar to that reported in literature (8); however, the FeS_{nano} spectrum although clearly different to the mackinawite spectrum is rather similar to the spectrum of greigite (8). This applies to features characteristic of the main peak, the shoulder feature and in the intensity of the pre-edge (Fig. 3).

Among these three features, the pre-edge (1s-3d) is the most reliable to provide information about the coordination environment of Fe and its oxidation state (9-13). The centroid of the pre-edge is unaffected by the resolution of data collection and is a good indication of the chemical environment (9). For oxides, any variation in the oxidation state of Fe (Fe^{II} and Fe^{III}) could be identified because the two species have centroids at different energies that are normally separated by ~1.4eV (9).

To fit the XANES data, we used the method developed for oxides and later applied to silicate glasses, hydrous melts, sulfides and oxide-sulfide mixtures (9, 10, 14, 15). These studies have demonstrated that the centroid of the pre-edge (which is related to the positions of the individual components) is a reasonably accurate to estimate the Fe^{II}/Fe^{III} ratio of an unknown sample composed of sulfides and oxides, when comparing the centroid position to those of the standards (15).

Comparison of the centroid values of FeS_{nano} and mackinawite (7112.8 vs 7112.7 eV) indicates a slightly higher proportion of Fe^{III} for the FeS_{nano} sample (Supplementary Fig. 10a). Furthermore, in supplementary Fig. 10b a shoulder feature less pronounced in FeS_{nano} than in

mackinawite spectrum could be observed. The higher energy of this feature in the spectrum for the FeS_{nano} compared to that of mackinawite indicates a slightly higher proportion of Fe^{III} present in FeS_{nano} and this is in accordance also with our XPS data (Supplementary Table 6). Additional marked differences in the fingerprint of the near-edge feature were also observed between the FeS_{nano} and mackinawite spectra, and the peak of the FeS_{nano} phase was positioned at higher energy compared to that of mackinawite, again consistently with the above analysis.

<u>EXAFS results</u>

Information about the local environment of Fe in the FeS_{nano} phase was obtained from analysis of the EXAFS portion of the XAS spectra. Our first approach was to compare the FeS_{nano} and mackinawite EXAFS spectra (Supplementary Fig. 11). In this preliminary analysis, we observed that the oscillations in the *k*-space of the FeS_{nano} spectrum were slightly shifted above $k > 5Å^{-1}$, with respect to the oscillations in the mackinawite spectrum. This suggests that the atomic structure of the FeS_{nano} phase is different than the mackinawite structure as manifested in the spectra between 5 and 9 $Å^{-1}$. The difference between both spectra becomes clearer when we plot the residual spectrum of mackinawite and FeS_{nano} (Supplementary Fig. 11b)

The second approach was to compare our experimental data to data for mackinawite and greigite in the theoretical models. In the mackinawite model, Fe is present in tetrahedral coordination while in the greigite model Fe is assigned both tetrahedral and octahedral environments. Thus we first fitted mackinawite as a reference and then tested this same approach for the FeS_{nano} spectrum. The fitting of the mackinawite EXAFS data to the mackinawite model was straightforward (Supplementary Table 10). The reduced χ^2= 7.04 and *R*-factor=0.01 indicated a very good fit in agreement with the model. Furthermore, ΔR (<0.1Å), ΔE_0 (<5 eV) and σ^2 were all within reasonable values. The interatomic distances for Fe-S of 2.23 Å and for Fe-Fe of 2.62 Å were also in a good agreement with the reported data (Fe-S = 2.24 Å and Fe-Fe = 2.63 Å or Fe-S = 2.23 Å and Fe-Fe = 2.60 Å (16, 17). Coordination numbers in the first shell (Fe-S) were 3.28 ± 0.12 and in the second shell 3.41 ± 0.65; however, these values must be used with caution as self-absorption problem in the data cannot be discarded given that the mackinawite spectrum had to be collected in fluorescence mode due to the small particle size or the freshly synthesized material. When this mackinawite EXAFS data was fitted to the to greigite model the results were far less

good as those obtained when fitting using the mackinawite model as the fit results for the second shell yielded extremely high σ^2 values. With these fits we could show that our experimental mackinawite data match well the mackinawite model from the literature.

For the fitting of the FeS_{nano} EXAFS spectrum, first we carried out a visual comparison with both the theoretical mackinawite and the theoretical greigite models before performing the actual fitting. The contribution of the paths was tested by plotting both these models along with the experimental data (Supplementary Fig. 12). Only the first two Fe-S and Fe-Fe paths were included in subsequent fits as they represent the most significant contribution to the models in the usable *k*-range. We observed that between the two models, the one built based on both the Fe-S and the Fe-Fe paths of the greigite structure allowed for a much better fit for the FeS_{nano} EXAFS data. In contrary, an adequate fit was only achieved for the model based on the mackinawite structure for the first Fe-S path. Nevertheless, we fitted the FeS_{nano} EXAFS data to both above-described models in order to better compare the results.

Results of these comparisons are presented in Supplementary Fig. 13 and Supplementary Table 11. *R*-factors and χ^2 yielded comparable results for both models (*R*-factor=0.005, reduced χ^2= 178 using the greigite model and *R*-factor=0.009 and reduced χ^2= 237 using the mackinawite model). Although, these parameters suggest a good fit of our experimental FeS_{nano} phase with both models, in the fit to the mackinawite model only the Fe-S path could be modelled. When the Fe-Fe path was included, the fit yielded a large σ^2 value and the reduced χ^2 increases considerably. In contrast, the fit to the model based on the greigite structure allowed the inclusion of both the Fe-S and the second Fe-Fe shell and this way the fit and the goodness-of-fit parameters were massively improved. These results indicate that the structure of the FeS_{nano} phase is more akin to the combined tetrahedral and octahedral environment of Fe that is typical for greigite, and distinctly different from that of mackinawite. These results are in full agreement with the results from the analysis of the XANES spectra.

The fit parameters obtained using the greigite model were both reasonable at ΔR (<0.1Å) and ΔE (< ±5 eV) and the value for σ^2 was similar (~ 0.006 Å) for the first and second shell. According to the greigite model, the first-shell has four S atoms around the central Fe atom, with interatomic distances of 2.23Å and in a second shell, two Fe atoms with 4.10Å bond distances. From the fit, coordination numbers (*N*) of 4.95 ± 0.19 for the first shell (Fe-S) and 2.09 ± 1.14 for the second

shell (Fe-Fe) were derived. It is worth noting however that as mentioned before for the mackinawite fit, the coordination number values may not be accurate as this parameter is subject to errors for samples there are characterized by very small particle sizes (18). This is the case for the FeS_{nano} phase that is made up of particles that are ~1-2 nm in size. Nevertheless, based on these fits we have demonstrated based on both the XANES and EXAFS analyses that the new phase FeS_{nano} is drastically different in structure than the synthesized mackinawite.

It is important to note that although the fitting was performed over a small *k*-range (2.8 and 6.5 $Å^{-1}$ for FeSnano and 3-10 $Å^{-1}$ for mackinawite), the number of independent points used for the fitting was twice the number of variables (Supplementary Table 10 and Table 11). Within these *k*-ranges two different absorber - scatterer distances (Fe-S and Fe-Fe) larger than 0.43 Å (ΔR) were resolved, which is above the resolution given by the *k*-range used in accordance with the rule of thumb in which $k_{max}- k_{min}>\pi /(2x\Delta R)$.

For over 30 years, there has been considerable debate over Fe-S bond lengths and coordinations in various Fe-S minerals (e.g., cubanite, greigite, chalcopyrite, pyrite and smythite; 19 - 22). All data seem to indicate a dependency between the valence of the Fe atom and the bond lengths (22). Mossbauer studies have shown that tetrahedral Fe in greigite has an oxidation state between 2+ and 3+; however, based on empirical calculations these oxidation states would yield longer Fe-S bond lengths than the actual 2.148 Å, and theoretically Fe^{+4} would yield a bond length of 2.144 Å (19, 21) close to 2.148 Å as in greigite (23). The increase in oxidation state is inferred to occur as a result of electron back-donation from S atoms yielding shorter bond lengths (24).

Transformation of FeS_{nano} to mackinawite

The new FeS_{nano} phase could be stable for months if kept at pH < 4.5 but upon addition of more NaHS, and thus an increase in pH transforms this new phase into mackinawite (Supplementary Fig. 3b). At pH between 4.5 and 5.0, the transformation leads first to a poorly ordered, also nanoparticulate, mackinawite as confirmed by diffraction and TEM analyses (Supplementary Fig. 3c-e) that showed agglomerates (Supplementary Fig. 3d) with *d*-spacings matching those for early mackinawite (Supplementary Table 3, e.g. 5.5 Å and reference (35). The *d*-spacings of this first mackinawite are still larger than those in the crystalline mackinawite (36),

which was our end product of the reactions when the pH was raised to 7. The XPS spectra of both the poorly ordered agglomerated mackinawite and the crystalline end-product mackinawite confirmed up to 30 % Fe^{III} in their structure (Supplementary Fig. 7; Supplementary Table 6), a value similar to the ferric iron content in the new FeS_{nano} phase. This is not surprising considering that the mackinawite transformed from the Fe^{III} containing precursor FeS_{nano}.

Supplementary Table 1. Titration experiments. Resulting solid samples were analyzed with XRD and imaged by TEM.

**Experiment**	**[Fe^{2+}], mM**	**[NaHS], mM**	**Parameter measured/min**
1	100	1000	pH 4-7, 9 min, 4.2 mL 1000 mM NaHS
2	100	500	pH 4-7, 33 min, 16 mL 500 mM NaHS
3	100	150	pH 4-7, 8 hours, 244 mL 150 mM NaHS
4	100	150	Same as above
5	100	150	Same as above

Supplementary Table 2. Diffusion experiments, and the reaction end pH; listed are also the analyses performed on each of the resulting solid products.

Experiments	pH	Analyses performed
1	4.3	XRD, TEM
2	4.3	XRD, TEM, Raman
3	4.1	XRD, Raman
4	4.1	First attempts for EXAFS
5	4.1	First XPS attempts, Raman, TEM
6	4.1	TEM
7	4.1	Raman
8	4.1	XPS
9	4.1	Raman
10	4.1	XRD
11	4.1	XANES, EXAFS
12	4.1	TEM, aqueous Fe analysis

Supplementary Table 3. *d*-spacings of lattice planes derived from FFT evaluations of multiple HR-TEM images acquired from different samples of the new FeS_{nano} phase synthesized by diffusion. In bold are values for the large *d*-spacings of the crystal lattices of the new FeS_{nano} phase that are not present in mackinawite.

Individual particle areas	FeS_{nano} *d*-spacing (Å)	Mackinawite *d*-spacing (Å)
1		1.8
2		2.3
3	**11.6**	
4	**11.9**	
5	**6.9**	
6	**11.2**	
7		2.2
8	**12.5**	
9		5.9
10	**9.8**	
11		4.4
12		2.8
13		3.0
14	**6.4**	
15	**9.1**	
16		3.0
17	**5.8**	
18		2.9
19		5.4
20		2.7

Supplementary Table 4. Raman vibrations from the FeS_{nano} phase produced through the diffusion method. The values in brackets correspond to the widths of the peaks and this difference can introduce variations in the absolute positions of the peak.

**Raman shift cm^{-1}**	**Vibration**	**Peak intensity**	**Reference**
105	Grease		
114	Grease		
121	Grease		
128	Grease		
148	S_2^{2-}	Medium	(37)
			(38)
162 (155-167)	S_n^{2-}	Small	(38)
170 (160-181)	S_2^{2-}, lattice vibrations	Medium	(39)
204 (170-247)	Fe-S, asymmetric stretching vibration	Medium, broad peak	(2)
215 (204-228)	Fe-S, asymmetric stretching vibration	Medium	(40)
228 (203-260)	S_n^{2-} n>8*	Small, broad peak	(40)
274 (233-317)	Fe-S S-S, S_n^{2-} n>8*	Medium, broad peak	(40)
323 (266-420)	S_n^{2-} n>8*	High, broad peak	(40)
378 (313-438)	S_n^{2-} n>8*	Medium, broad peak	(40)
463 (368-532)	S_n^{2-}, asymmetric stretching vibration	High broad	(40)
494 (475-511)	Grease		
553 (475-623)	S_2^{2-}, Grease	Small, broad peak	(37)
668 (528-775)	Grease	High broad	
703 (656-755)	Grease	Medium broad	

*Polysulfides

Supplementary Table 5. Raman vibrations from freshly synthesized mackinawite end product at pH 7 using the titration method. Polysulfide bands were not detected in these samples, however if present in minor amounts such bands could overlap with or form minor shoulders on the high intensity Fe-S vibrations.

**Raman shift cm^{-1}** <br>**This study**	**Vibration**	**Peak**	**Reference**
210	Fe-S, asym. stretch.	High intense	(208 cm^{-1}, 214 cm^{-1}) (2, 3)
272	Fe-S, sym. stretch.	Medium, intense	(281* cm^{-1}, 283* cm^{-1}, 298* cm^{-1}) (2); (282 cm^{-1}) (3)
386	Fe-O	Small, broad	(386 cm^{-1}) (3)
482	Fe-O, grease	Small, broad	
560	Grease	Small, broad	
638	Grease	Small, broad	

asym=asymmetric, stretch=stretching, sym= symmetric.

*Depending on ageing from 15 minutes to 12 days

Supplementary Table 6. XPS Fe and S speciation data for the FeS_{nano} phase and mackinawite including two analyses of post-argon etching of the FeS_{nano}. Binding energies were correct at a ± 0.1eV resolution. The surface composition is a semi-quantitative measure (±10%) calculated from the peak areas (Fe 2p and S 2p).

**Sample**					**% (±10%)**
	**Fe 2p$_{3/2}$**				
**FeS_{nano}**	707.9	1.8	58.8	Fe^{II}-S	58
	709.8	1.8	30.7	Fe^{III}-S	42
	711.9	1.8	10.5	Fe^{III}-S	
**FeS_{nano}**	708.0	0.9	57.3	Fe^{II}-S	57
**(After etching)**	710.0	0.9	29.0	Fe^{III}-S	43
	712.3	0.9	13.7	Fe^{III}-S	
**Mackinawite**	706.9	1.0	35.6	Fe^{II}	73
	707.7	1.0	20.8	Fe^{II}	
	713.4	1.0	1.9	Fe^{II} satellite	
	708.5	1.0	14.7	Fe^{II}	
	712.2	1.0	3.5	Fe^{III}	27
	709.4	1.0	10.5	Fe^{III}	
	711.2	1.0	5.2	Fe^{III}	
	710.2	1.0	7.8	Fe^{III}	
	**S 2p$_{3/2}$**				
**FeS_{nano}**	161.1	0.8	44.7	Monosulfides	62
	161.8	0.8	22.4	Monosulfides	
	162.9	0.8	14.9	Disulfides	14
	163.2	2.2	18.1	Polysulfides	17
**FeS_{nano}**	161.2	0.9	32.9	Monosulfides	68
**(After etching)**	161.9	0.9	35.4	Monosulfides	
	162.7	0.9	13.0	Disulfides	13
	163.7	1.6	18.7	Polysulfides	19
**Mackinawite**	160.9	0.7	24.4	Monosulfides	53
	161.6	0.7	39.0	Monosulfides	
	162.2	2.6	36.6	Polysulfides	31

Supplementary Table 7. XPS Fe reference binding energies (eV).

**Mineral**						
**Pyrrhotite**	Fe_7S_8, vacuum	706.5<br>707.5<br>708.4			713.3	(*41*)
**Pyrite synthetic**	FeS_2	706.7				(*42*)
**Pyrite synthetic**	FeS_2	707.5				(*43*)
**Pyrrhotite**	$Fe_{0.89}S$	707.0				(*44*)
**Pyrite**	FeS_2	707.0				(*5*)
**Pyrrhotite synthetic**	$Fe_{1-x}S$	707.5				(*45*)
**Troillite, Pyrrhotite**	FeS, $Fe_{1-x}S$	706.8<br>707.8<br>708.5	709.2<br>710.3<br>711.3<br>712.2	710.3<br>711.3<br>712.4<br>713.5	713.6	(*46*)
**Mackinawite synthetic**	FeS, $Fe_{1+x}S$	706.8<br>708.5	709.6<br>710.6<br>711.6			(*7*)
**Mackinawite synthetic**	$Fe_{1+x}S$	707.6	709.1	711.6		(*47*)
**Mackinawite synthetic**<br>**Greigite**	$Fe_{1+x}S$<br>Fe_3S_4	706.8	708.5<br>709.6<br>710.6<br>711.6	710.7		(*48*)
**Mackinawite synthetic**	$Fe_{1+x}S$	706.4<br>707.3<br>708.2	709.2<br>710.4<br>711.4<br>712.7		713.3	(*48*)

Supplementary Table 8. XPS Sulfide reference binding energies (eV).

**Mineral**	**Nominal Composition**	**Mono (S^{2-})**	**Mono (S^{2-})**	**Di (S_2^{2-})**	**Poly (S_n^{2-})**	**Reference**
Sulfur	S(0)				163.7	(49)
Pyrite	FeS_2				163.9	(50)
Pyrite	FeS_2			162.3		(51)
Pyrite	FeS_2			162.4		(49)
Pyrite	FeS_2			162.5		(5)
Pyrite	FeS_2			162.5		(52)
Pyrrhotite	$Fe_{0.89}S$	161.1				(44)
Sphalerite	ZnS	161.2				(53)
Galena	PbS	160.7				
Pyrrhotite	Fe_7S_8	161.2				
Mackinawite	FeS		161.8			(54)
Pyrrhotite	$Fe_{1-x}S$	161.1				(45)
Sphalerite	ZnS	161.6				(55)
Galena	PbS	160.6				(52)
Tin(II) Sulfide	SnS	161.1				(56)
Manganese (IV) disulfide	MnS_2		161.9			(53)

Note: Metal-SO_4^{2-} species binding energies are ~ 169 eV, so no sulfate species were neither present in FeS_{nano} nor in mackinawite

Supplementary Table 9. XAS pre-edge peak analysis for mackinawite, FeS_{nano} and the analyzed references.

Sample	Centre of gravity	Height	FWHM	Area	Total Integrated Area	Centroid	R^2	Reduced χ^2
Mackinawite	7112.4	0.0929	1.8	0.18	0.26	7112.7	0.999	2.78E-06
	7113.3	0.0440	1.8	0.08				
FeS_{nano}	7112.4	0.0374	1.4	0.06	0.09	7112.8	0.999	3.02E-07
	7113.4	0.0260	1.4	0.04				
Magnetite (Fe_3O_4)	7113.1	0.0127	1.3	0.02	0.10	7114.0	1.000	5.51E-08
	7113.9	0.0439	1.3	0.06				
	7114.9	0.0165	1.3	0.02				
Hematite (Fe_2O_3)	7113.4	0.0251	1.3	0.03	0.09	7114.1	0.999	1.51E-07
	7114.7	0.0364	1.3	0.05				
	**7115.9***	**0.0125**	**1.3**	**0.02**				
	**7117.3***	**0.0064**	**1.3**	**0.01**				
Wustite (FeO)	7111.7	0.0245	1.3	0.03	0.12	7112.7	0.998	7.48E-07
	7113.7	0.0299	1.3	0.04				
	7112.6	0.0314	1.3	0.04				

* Components not included in the calculation of the centroid as they are not predicted by the crystal-field theory in the 1S-3d transition, the arise from high spin Fe^{III} in octahedral symmetry (*17*)

Supplementary Table 10. EXAFS fitting parameters derived from fitting the mackinawite spectrum; the degeneracy of each path was based on the theoretical models for mackinawite and greigite.

**Mackinawite**	
N_{ind}	16
N_{var}	5
**Reduced** χ^2	7
***R*-factor**	0.01
ΔE_0 **(eV)**	-2.78 ± 0.94
S_0^2	0.80
	**Sulfur**
N	3.28 ± 0.12
ΔR	-0.0212 ± 0.0076
R **(Å)**	2.235 ± 0.003
σ^2 **(Å^2)**	0.007 ± 0.001
	**Iron**
N	3.41 ± 0.65
ΔR	0.0236 ± 0.0158
R **(Å)**	2.621 ± 0.003
σ^2 **(Å^2)**	0.022 ± 0.001

Supplementary Table 11. EXAFS fitting parameters derived from fitting the FeS_{nano} spectrum. The degeneracy of each path in the mackinawite model was taken from the theoretical structure, while the paths in the greigite model were derived directly from the fit.

	**Mackinawite model**	**Greigite model**
N_{ind}	8	8
N_{var}	4	4
**Reduced** χ^2	237	178
***R*-factor**	0.009	0.005
S_0^2	0.80	0.80
ΔE_0 **(eV)**	-1.4 ± 1.3	-0.9 ± 1.2
	**Sulfur**	
N	5.08 ± 0.80	4.95 ± 0.19
R **(Å)**	2.225 ± 0.001	2.225 ± 0.001
σ^2 **(Å^2)**	0.006 ± 0.001	0.006 ± 0.001
	**Iron**	
N	-	2.09 ± 1.14
R**(Å)**	-	4.104 ± 0.01
σ^2 **(Å^2)**	-	0.006 ± 0.001

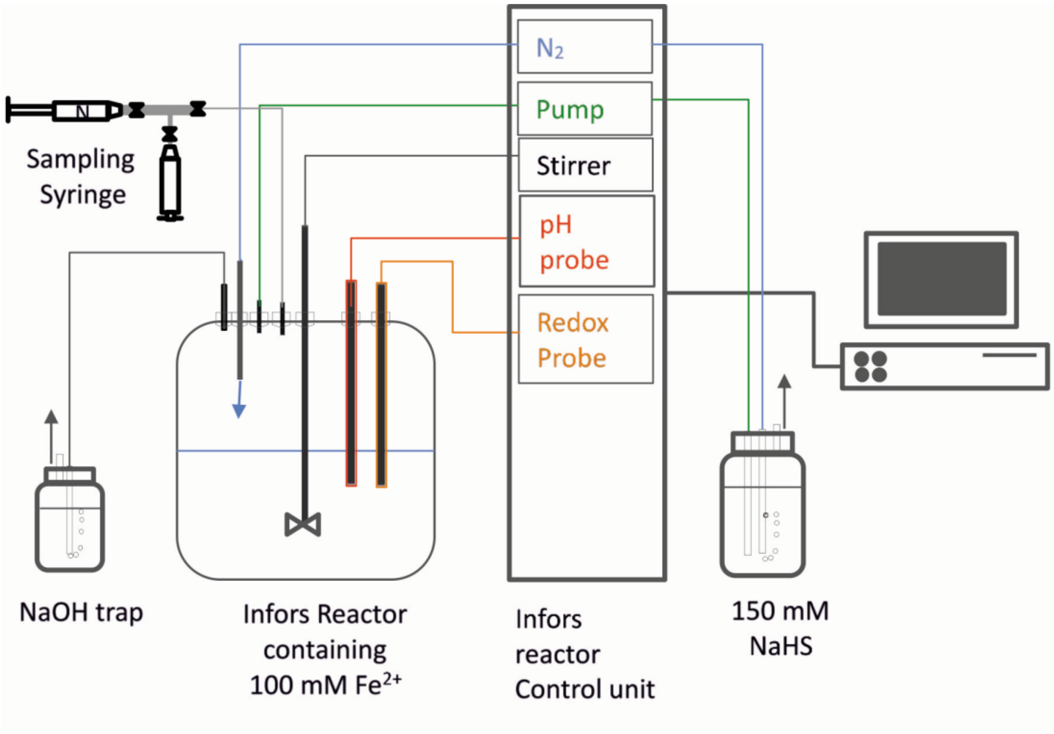

Supplementary Figure 1. Schematic representation of the titration experimental set up and the controlled NaHS dosing through the automated unit of the Infors reactor control unit. All initial Fe^{2+} and NaHS solutions were O_2-free and all preparations were done under anaerobic conditions which were maintained inside the reactor through a positive pressure of O_2-filtered N_2 gas (99.999%) throughout the whole length of each experiment. NaHS was slowly added to the Fe^{2+} solution by using a precision metering pump. The pH, Eh and injected volume of NaHS were recorded every minute.

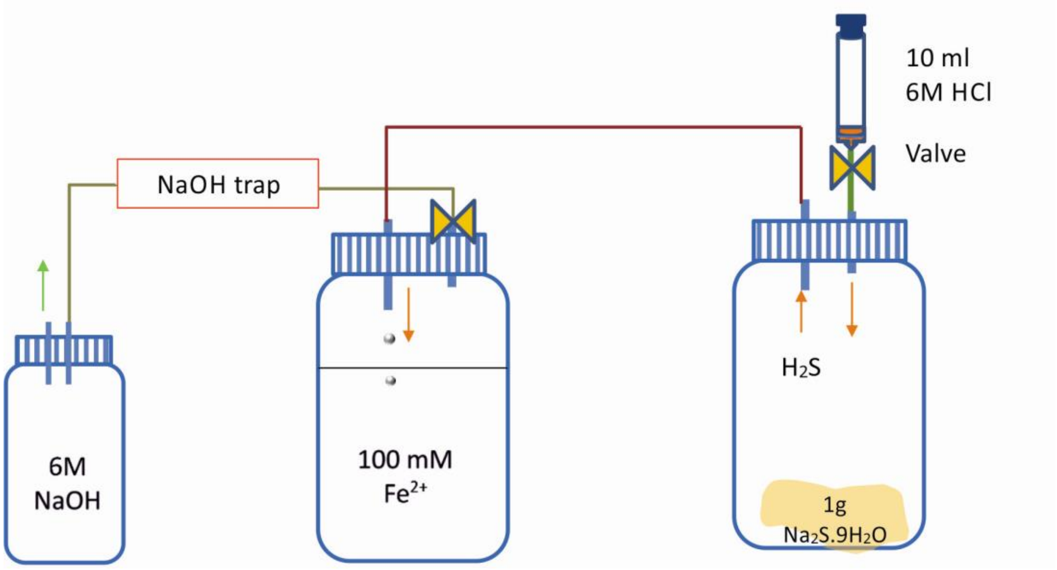

Supplementary Figure 2. Schematic drawing of the experimental set up used in the diffusion experiments. The experimental reactor (middle) contained a 100 mM Fe^{2+} solution, while the reactive H_2S gas was produced through the reaction of solid $Na_2S.9H_2O_{(s)}$ crystals and liquid HCl that was injected into the right hand bottle and then allowed to diffuse slowly into the experimental reactor; a NaOH trap was added to the system to capture any excess H_2S gas.

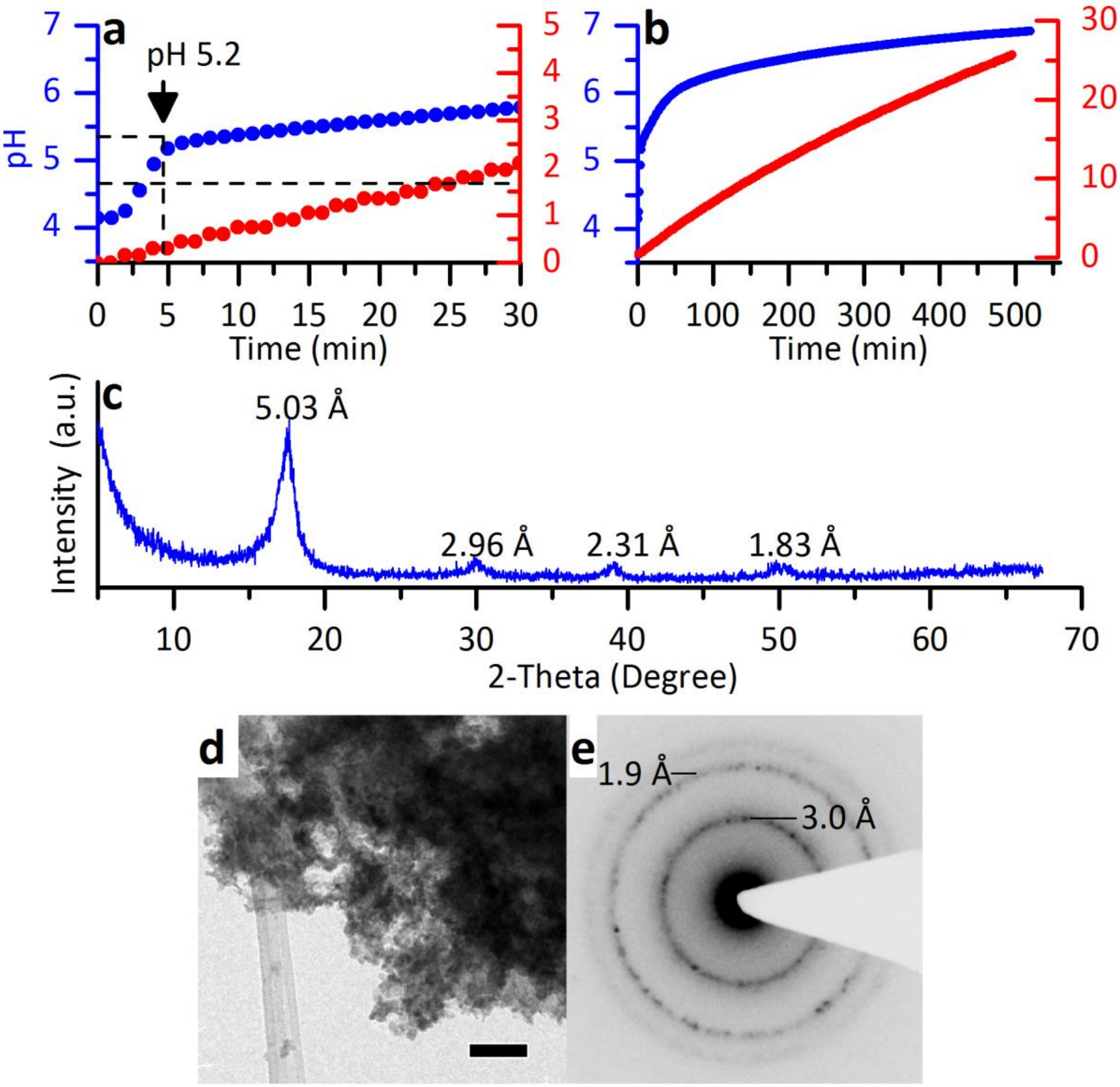

Supplementary Figure 3. Combined results from the titration experiments. This figure complements the data presented in Fig. 1 and the associated discussion in the main text. (**a**) Titration profile showing the increase in pH (blue) and addition of NaHS (red) between pH 4.1 and 5.5; 2 mM of NaHS were needed to reach a pH value just of ~ 4.3 at which point the formed, slightly greying precipitates were separated from the solution; (**b**) full titration profile up to pH 7.0 showing the full 9 hours of a titration experiment; (**c**) XRD of poorly crystalline mackinawite collected from a titration experiment once the pH of 5.2 was reached; (**d**) medium resolution TEM image (scale bar 100 nm) of mackinawite agglomerates observed at pH 5.2 and **e**) the corresponding selected area electron diffraction pattern showing the main mackinawite *d*-spacings (Supplementary Table 3); important to note is the absence in both **c** and **e** of the large *d*-spacings that are typical for the new FeS_{nano} phase (Fig. 1, main document).

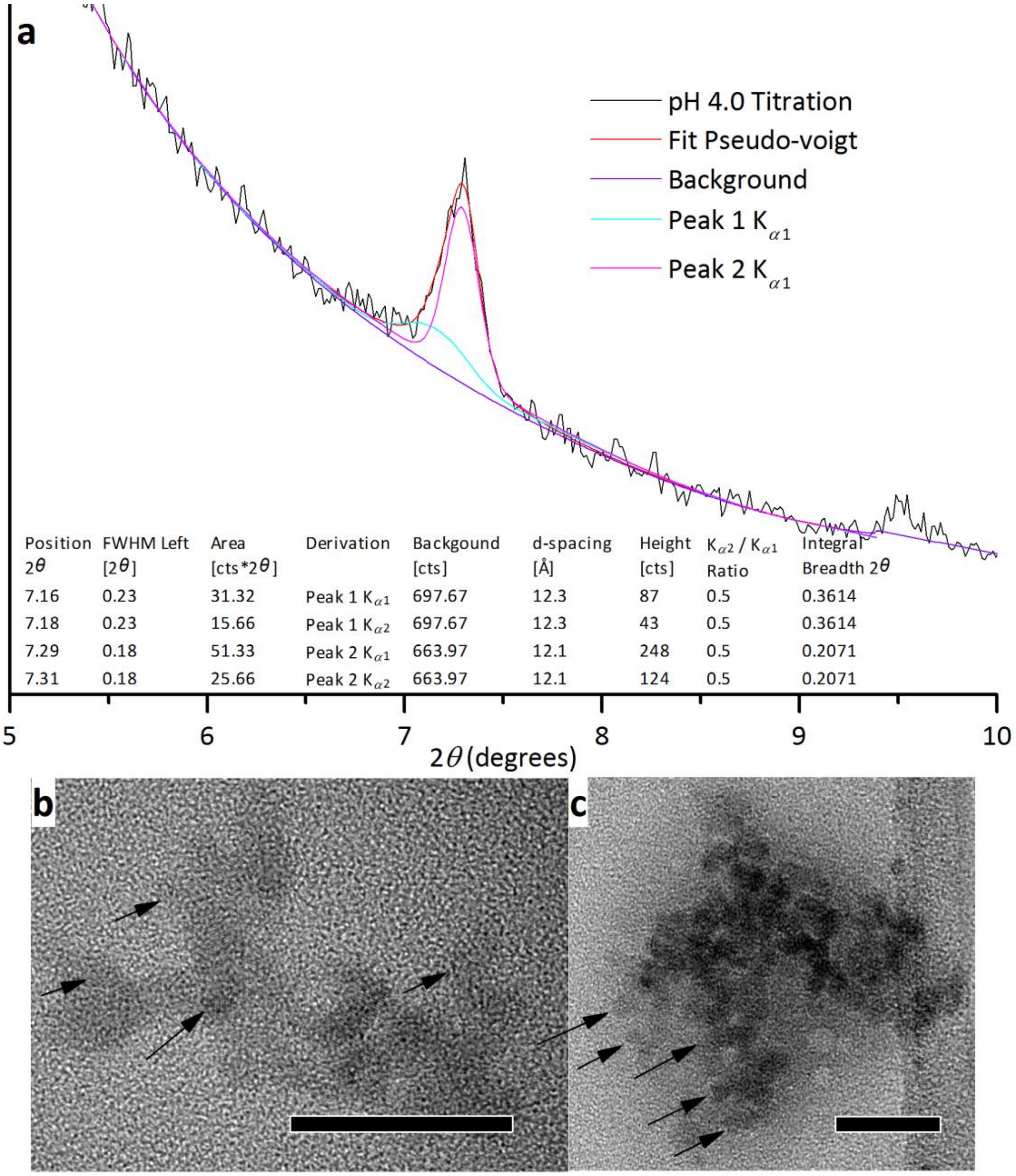

Position 2θ	FWHM Left [2θ]	Area [cts*2θ]	Derivation	Backgound [cts]	d-spacing [Å]	Height [cts]	$K_{\alpha2}$ / $K_{\alpha1}$ Ratio	Integral Breadth 2θ
7.16	0.23	31.32	Peak 1 $K_{\alpha1}$	697.67	12.3	87	0.5	0.3614
7.18	0.23	15.66	Peak 1 $K_{\alpha2}$	697.67	12.3	43	0.5	0.3614
7.29	0.18	51.33	Peak 2 $K_{\alpha1}$	663.97	12.1	248	0.5	0.2071
7.31	0.18	25.66	Peak 2 $K_{\alpha2}$	663.97	12.1	124	0.5	0.2071

Supplementary Figure 4. (**a**) Example of a peak fitting analysis of the XRD peak at ~12 Å from a titration experiment. Fitting in High Score Plus to a Pseudo-Voigt profile revealed two peaks, peak 1 (7.16 2θ) and peak 2 (7.29 2θ) from the contribution of Cu $K_{\alpha1}$ are shown in the figure. Crystallite size of ~23 nm and ~44 nm were derived from the fit, which is far larger than the 2 nm quantified from TEM likely reflecting the self-assembly or fusion of individual particles to form larger agglomerates but not their transformation; (**b**) This is confirmed by seemingly self-assembled ~ 2 nm individual nanocrystals through attachment and progression to form a bigger agglomerate as seen in TEM (scale bar 20 nm); (**c**) bigger agglomerate showing attachment of smaller crystals (scale bar 20 nm).

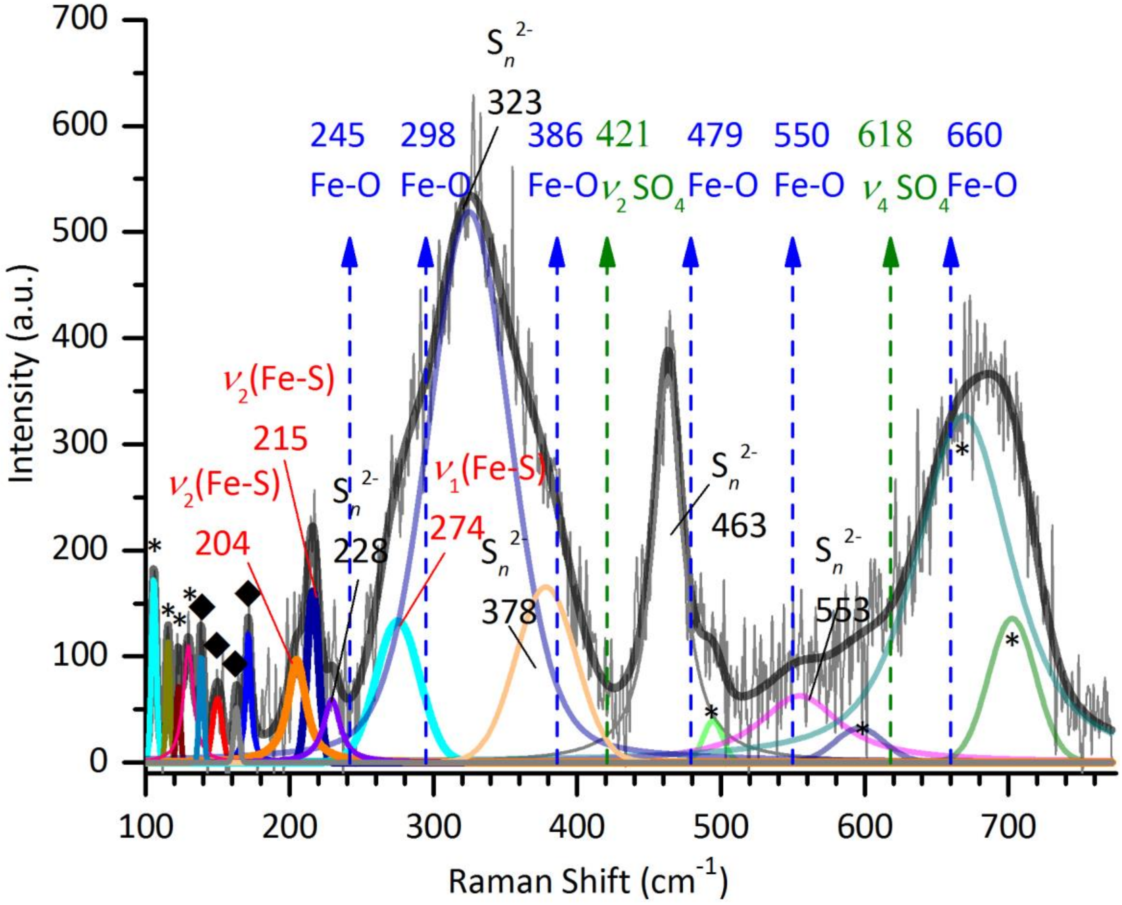

Supplementary Figure 5 Raman spectra for the FeS_{nano} showing the peak assignments. The blue dotted lines on the plot indicate the positions of the peaks for Fe-O species as known for α-FeOOH (245, 298, 386, 479, 550 and 660 cm^{-1}) (57); however, in our FeS_{nano} spectrum the main vibrations at 245 and 386 cm^{-1} are missing and thus no Fe-O peaks are present. The position for where the peaks for ν_2 and ν_4 for SO_4 species would be (at 421 and 618 cm^{-1}) are also shown by the green dotted arrows, yet they are clearly missing in this FeS_{nano} spectrum. The asterisks (*) in this spectrum refer to peaks from the grease used to seal the sample between the glass slides, while the diamonds (♦) pinpoint the short-chain polysulfide peaks identified in FeS_{nano} (see main text as well as Fig. 2a and Supplementary Table 2). a.u., arbitrary unit.

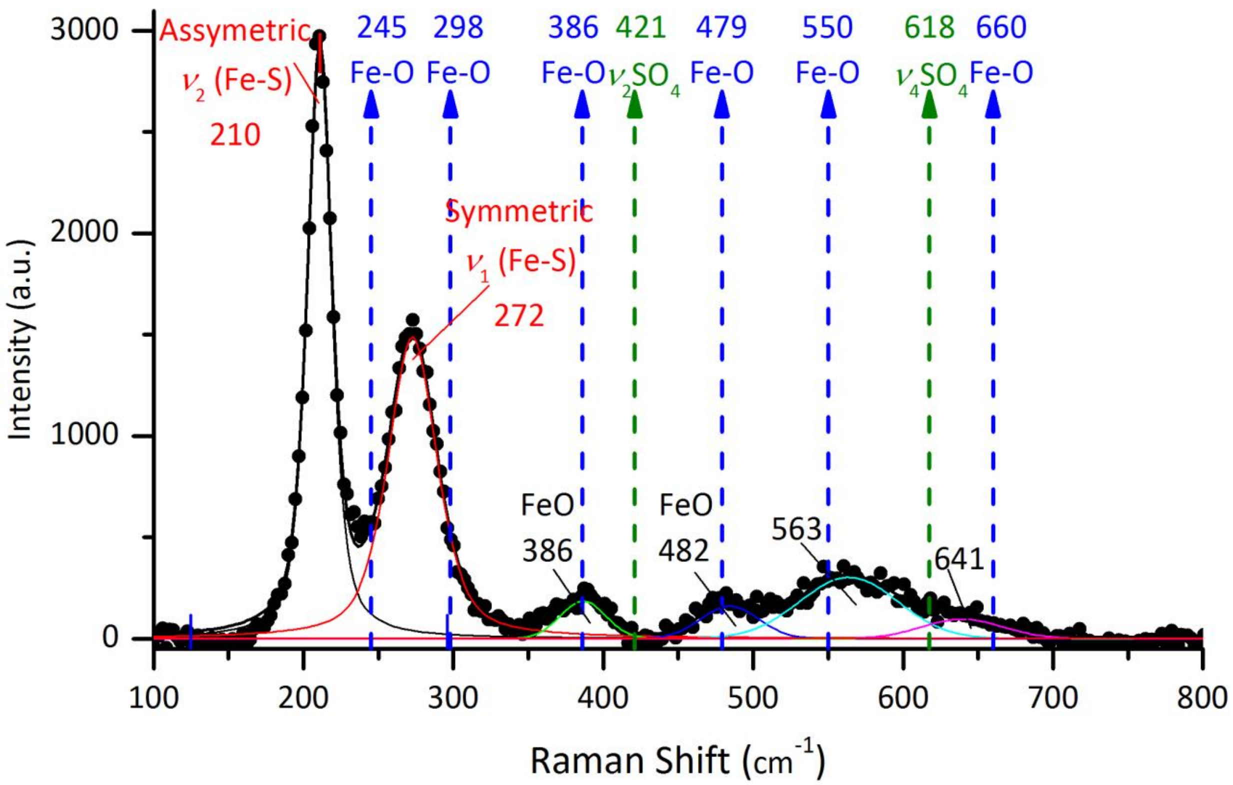

Supplementary Figure 6 Raman spectra of mackinawite collected from a titration experiment once the pH reached the end value at 7. The sample was analyzed using the 473 nm laser and five single spectra, each collected for 150 seconds were co-added. This sample was pure mackinawite as also cross-confirmed by XRD and TEM analyses (Supplementary Fig. 3). The Raman spectra shows two main peaks at 208 and 272 cm^{-1}, which are both characteristic for Fe-S vibrations in mackinawite, although the latter peak is shifted compared to literature values for synthetic mackinawite (2); 281 cm^{-1} for $Fe^{II}/S=3/2$, 283 cm^{-1} for $Fe^{II}/S=1$). A shift in the second characteristic peak has been suggested to be likely a consequence of aging and drying (i.e., the peak appears at 298 cm^{-1} when mackinawite was aged for 12 days under an argon atmosphere) (2). The spectra also shows very weak and highly broad bands at 386 and 482 cm^{-1} which could be interpreted as possible air oxidation of mackinawite. However, when the positions of possible Fe-O vibrations (marked with blue arrows) in other phases compared no match was found; similarly vibrations related to SO_4^{2-} were also absent (green arrows). a.u., arbitrary units.

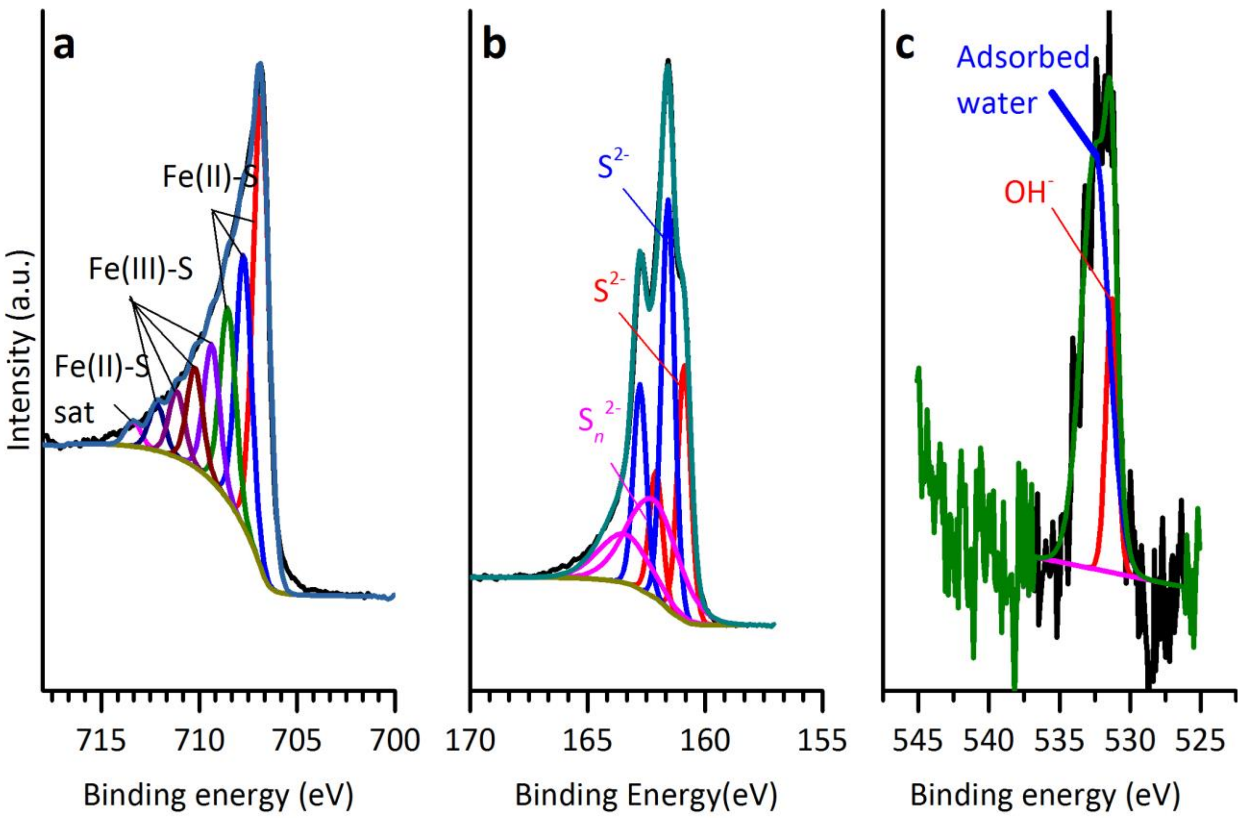

Supplementary Figure 7. XPS spectra of mackinawite solids collected from the solution at pH 5.5. (**a**) Fe $2p_{3/2}$ spectrum, (**b**) S $2p_{3/2}$ spectrum and (**c**) O 1s spectrum of mackinawite. Monosulfides (53%) and polysulfides (31%) are present in mackinawite, disulfides were not detected (Supplementary Table 6). Similarly to the FeS_{nano}, our mackinawite XPS data was best fitted by using two doublets for monosulfide species; however the overall fingerprint is very different to the FeS_{nano} spectrum. The polysulfides in mackinawite is highly different than that in the FeS_{nano} phase and this could be the result of aging of the mackinawite (2). Likewise, Fe^{II}-S and Fe^{III}-S species are present in mackinawite. Our mackinawite spectrum, as well as the fits and the identified species are highly similar to the few XPS spectra available in the literature (7, 47, 48, 58); however, there are some notable differences that could stem from the fact that all reported XPS data so far in the literature were collected from synthetic mackinawite samples that were produced through highly different experimental methods (different titrations, diffusions or chemical routes) and so far reproducibility in the characteristics of the mackinawite products through this different routes could not be demonstrated. We assert that as observed in our Raman and XPS spectra (Fig. 2, Supplementary Figs 5-7), the spectral fingerprints could be affected by aging, crystallization and local disorder within the mackinawite structure. Finally, it is worth noting that the O 1s spectrum from both the FeS_{nano} and mackinawite samples (Fig. 2d and Supplementary Fig. 6c) were best fitted with two peaks at 532 and 531 eV. These peaks have been associated with adsorbed water and hydroxyl groups, but importantly the missing peak (i.e., shoulder) at 529 eV is a clear confirmation of a lack of air oxidation of our samples (59). a.u., arbitrary unit.

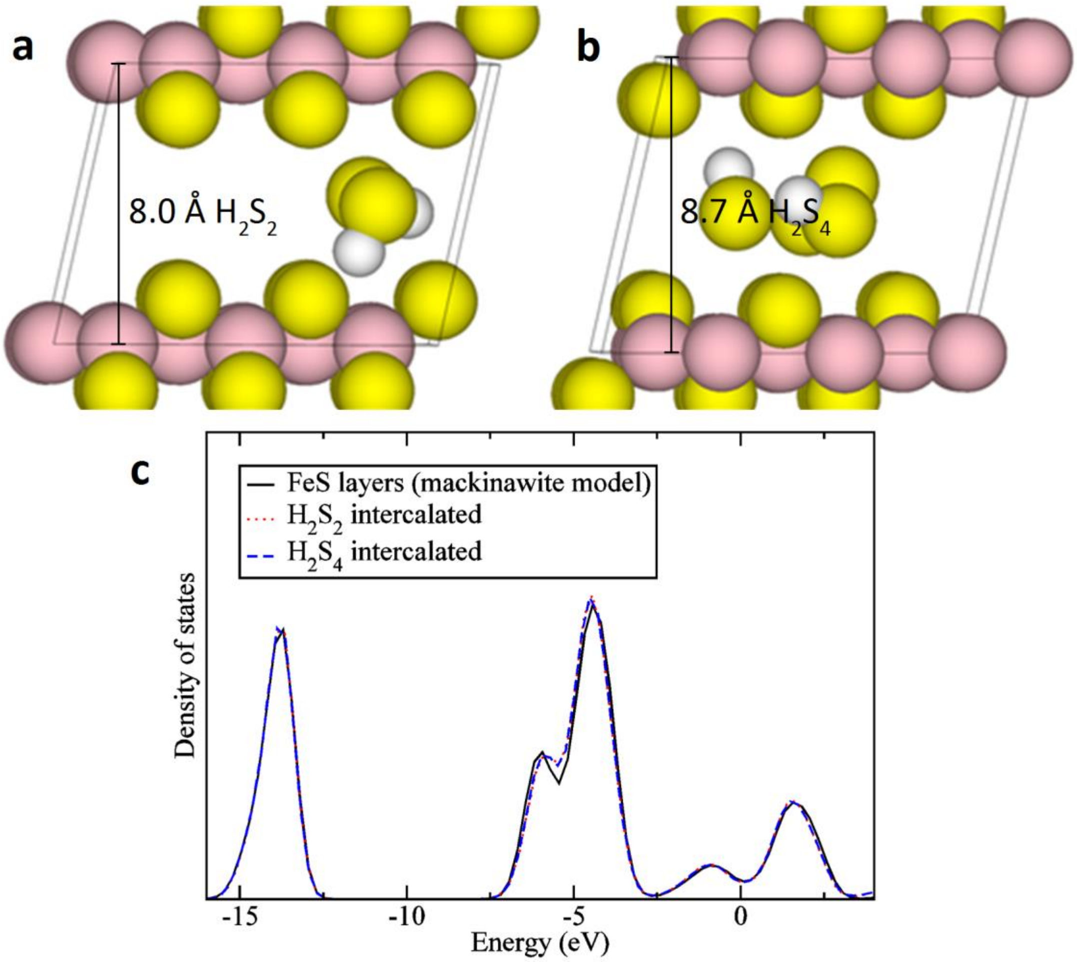

Supplementary Figure 8. DFT relaxed structures of H_2S_2 (a) and H_2S_4 (b) intercalated between mackinawite-structured Fe-S layers. The final supercells are also marked. Colour code: Fe pink, S yellow, H white; (c) Electronic densities of states of Fe-S layers with intercalated H_2S_2 and H_2S_4 projected onto the S atoms at the corners of the FeS_4 tetrahedra and compared to the Fe-S layered structure without polysulfides. The similarity between the electronic structures indicates that only non-bonded interactions are present (35). The Fermi energy is set to zero**.**

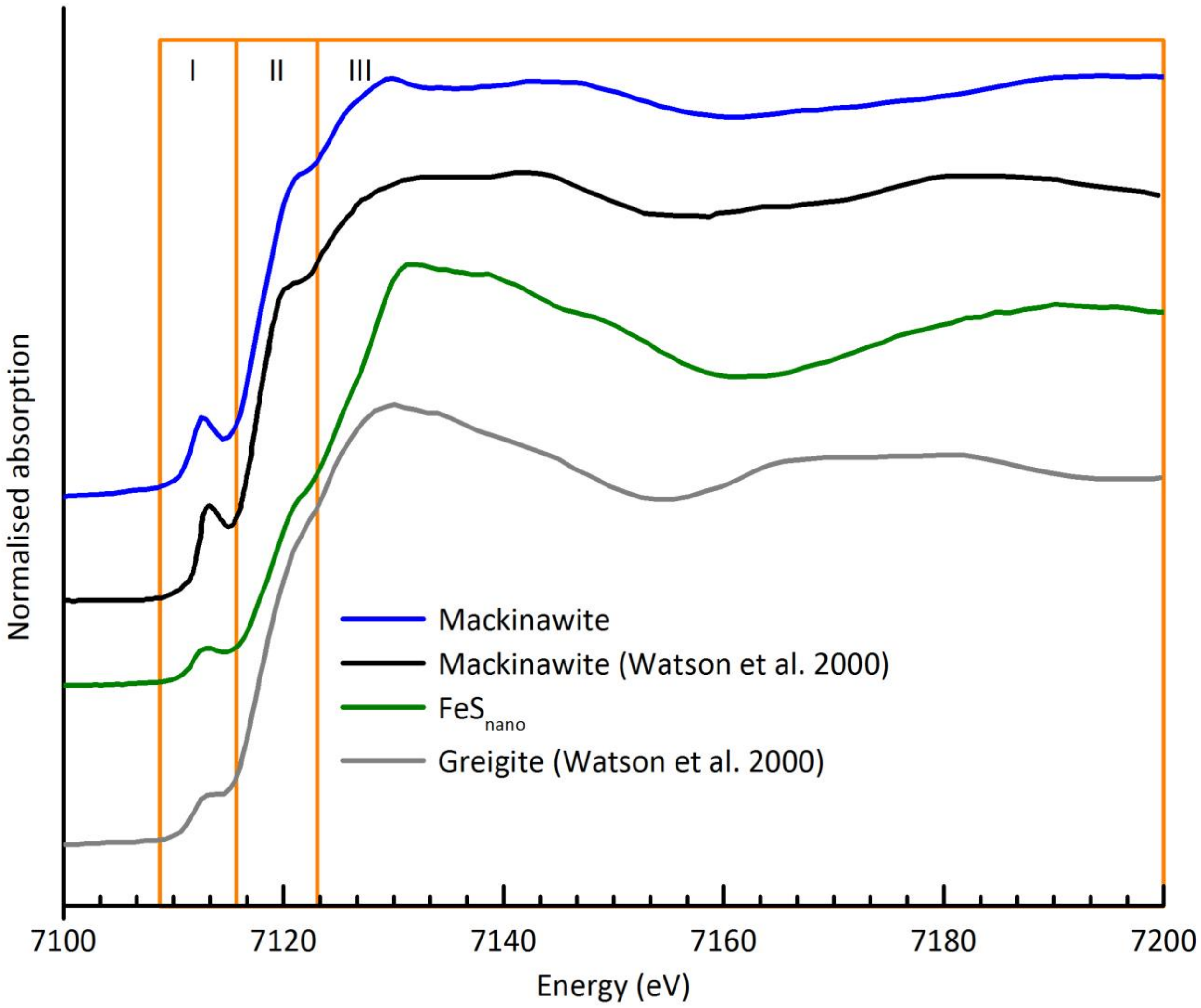

Supplementary Figure 9. Fe *K*-edge XANES spectra from FeS_{nano} and mackinawite. The spectral features (I) a pre-edge, (II) an edge jump and (III) a near-edge are indicated. Furthermore, the Fe *K*-edge XANES spectra for mackinawite and greigite from the literature are also shown (8). The mackinawite spectrum from our study is very similar in most of the features to the reported in literature (8). This plots clearly shown that the FeS_{nano} and the mackinawite spectra are different, in that the pre-edge in the FeS_{nano} spectrum is less pronounced and there is a clear difference in the shape of the main peak of the spectra; this latter feature and the shoulder are similar to the spectrum of synthetic greigite (8).

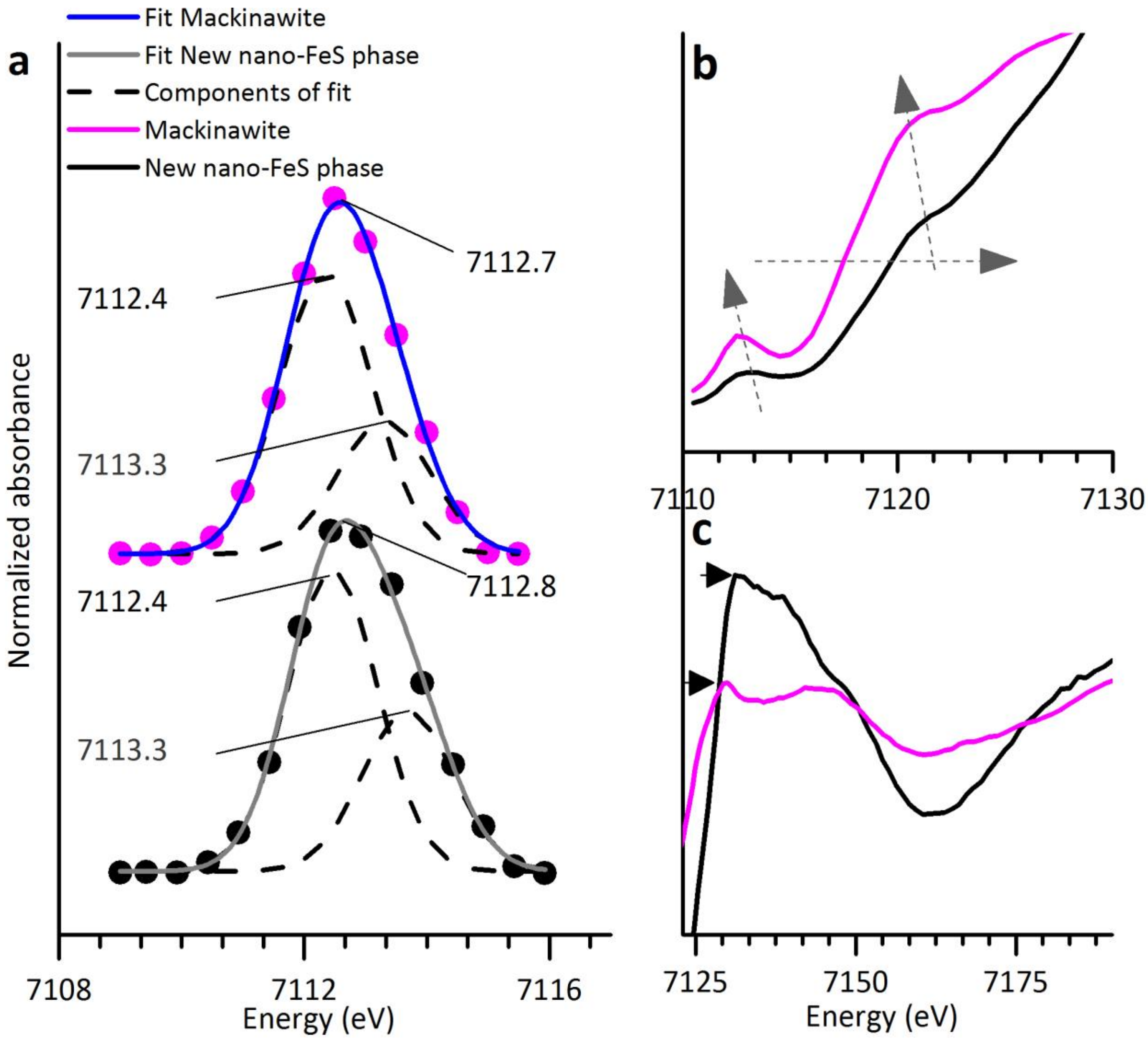

Supplementary Figure 10. Fe K-edge XANES features. (**a**) Pre-edge region showing the fit components (lines) to data points from mackinawite (blue) and FeS_{nano} (grey); (**b**) shows the pre-edge and the increase in the edge jump and (**c**) the main peak. The centroid in the pre-edge feature for both, FeS_{nano} and mackinawite, is positioned at higher (7112.7 eV) energies than typical ferrous compounds at 7112.1 eV indicating the presence of some Fe^{III} in both structures. As seen in (**b**) the increase in the edge jump is shifted towards higher energies for FeS_{nano} than for mackinawite, and this is also related to the higher proportion of Fe^{III} present in FeS_{nano} as also evidenced in our XPS data (Supplementary Table 6). In the near-edge region (**c**), there are clear differences in the shape of the main peak between FeS_{nano} and mackinawite, and again the peak of the FeS_{nano} phase is positioned at a higher energy, consistent with the presence of relatively more Fe^{III} in its structure.

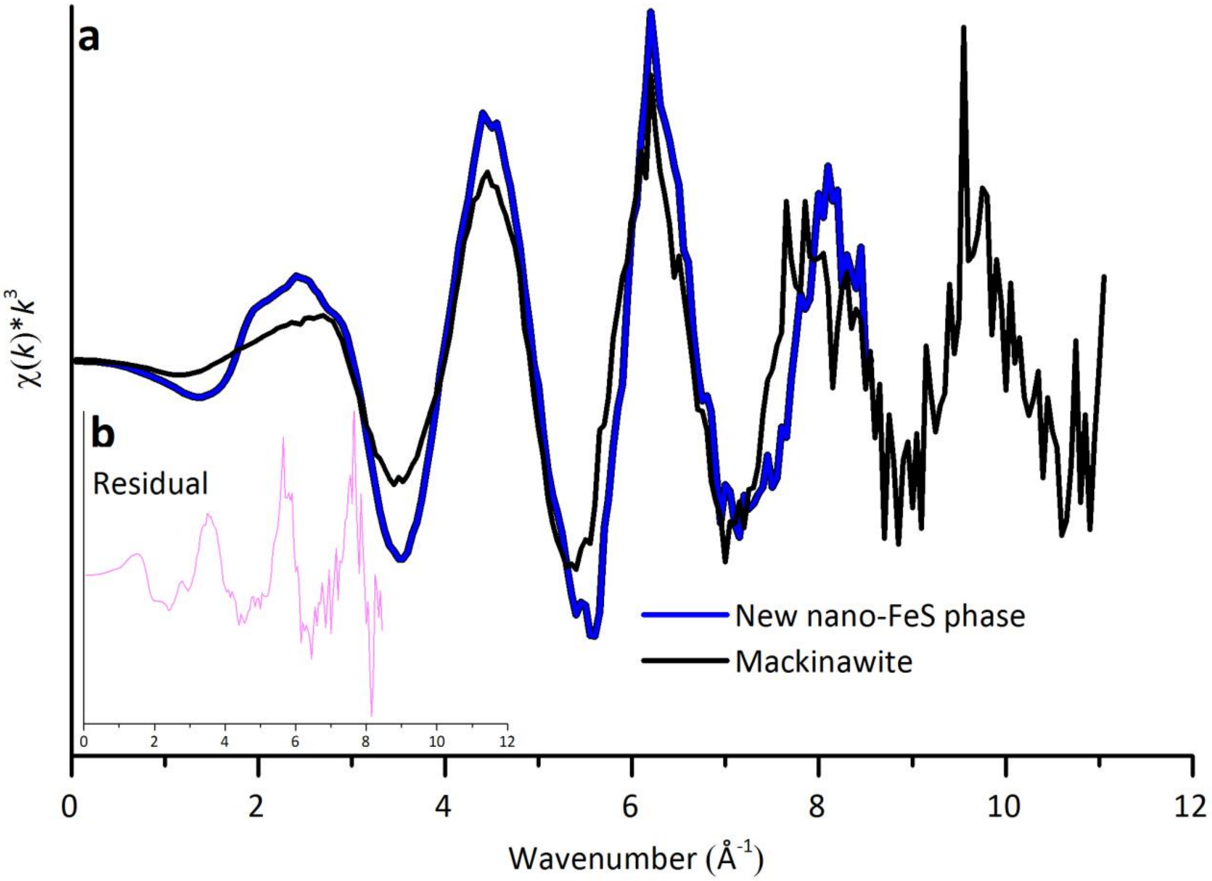

Supplementary Figure 11. EXAFS data acquired from FeS_{nano} and mackinawite. (**a**) Comparison between the EXAFS $\chi(k)$ data for FeS_{nano} and mackinawite; (**b**) difference EXAFS $\chi(k)$ spectrum for mackinawite minus FeS_{nano} evidencing the differences between both spectra. Comparing the two spectra, reveals that the oscillations above $k > 5Å^{-1}$ are shifted indicating that the atomic structure of FeS_{nano} is different from that of mackinawite and the shift becomes clearer when we plot the residual spectrum of mackinawite minus FeS_{nano}, which does not yield a flat line.

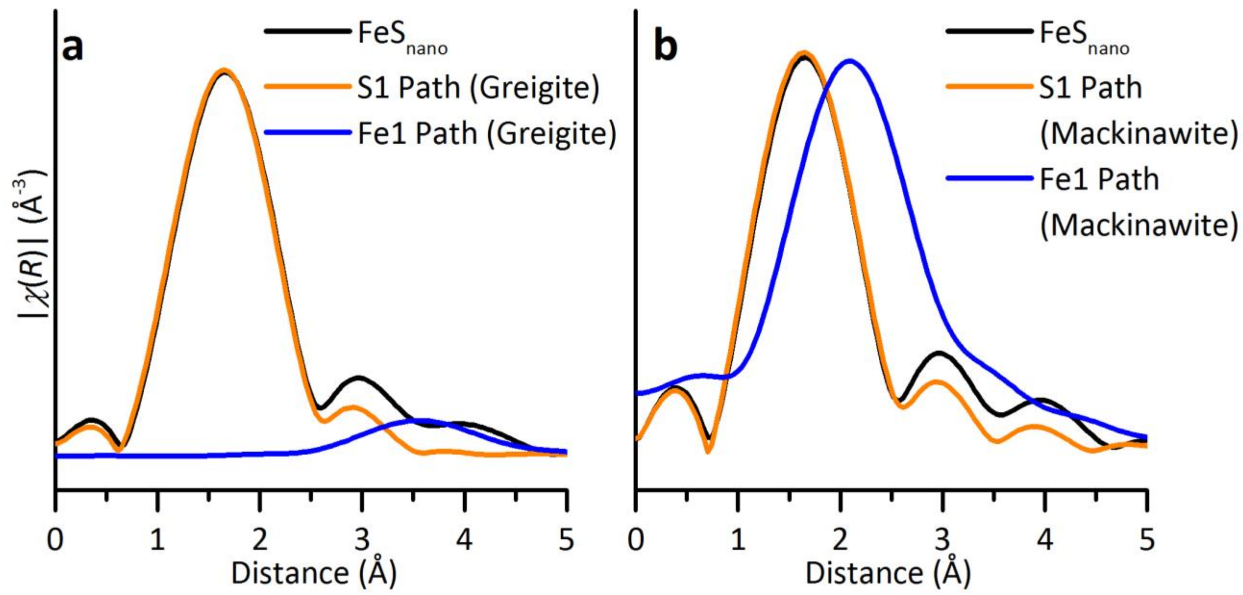

Supplementary Figure 12 Fe-S and Fe-Fe paths contribution to the model for (**a**) greigite and (**b**) mackinawite. The Fourier transform of the FeS_{nano} EXAFS data (black lines) was compared with the theoretical models for mackinawite and greigite before fitting. The contribution of the paths of each model was tested by plotting them along with the experimental data. Only the first two paths Fe-S and Fe-Fe were included in the subsequent fit as they were the most significant in the usable *k*-range; however, for mackinawite only the Fe-S path could be included.

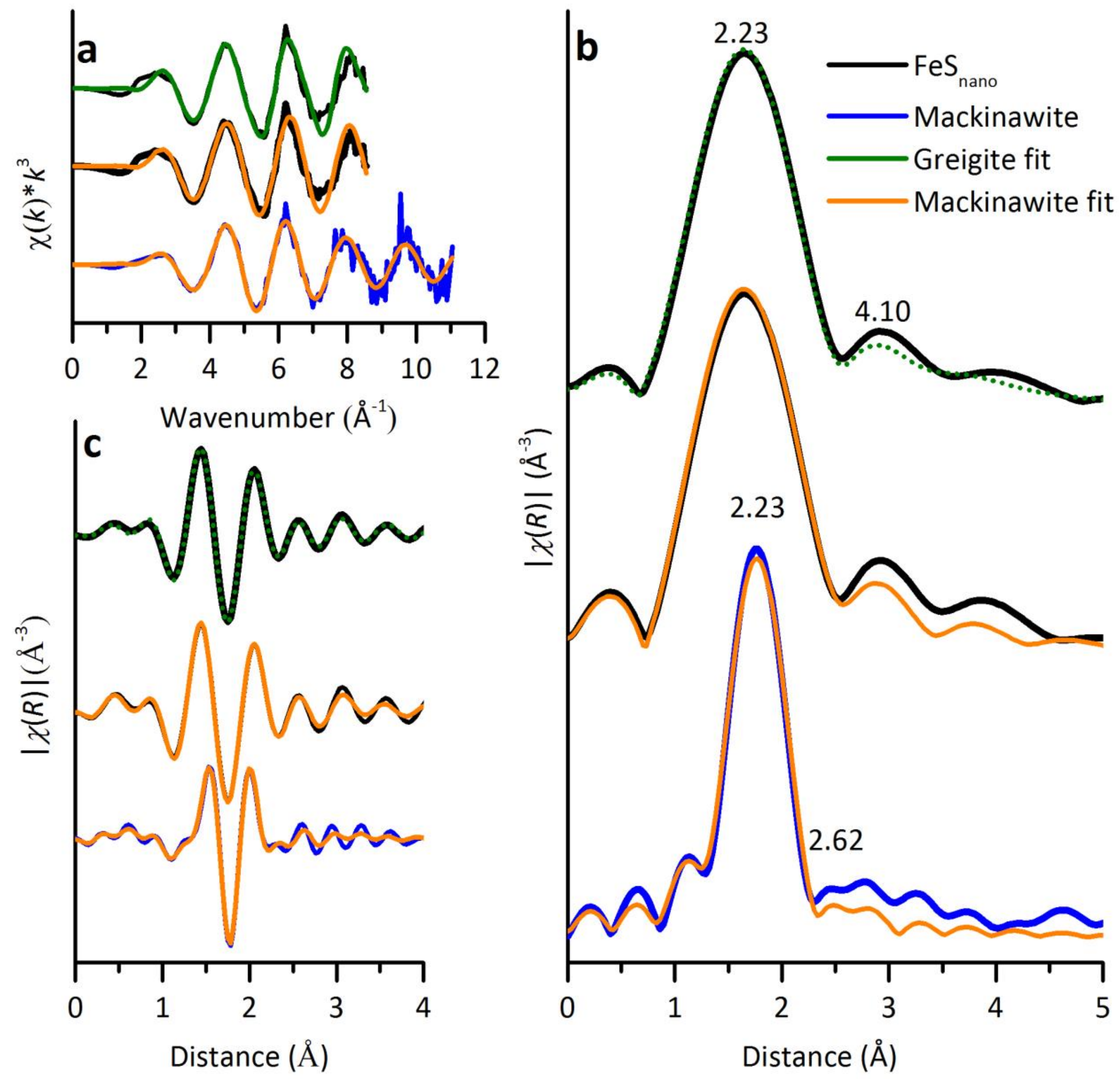

Supplementary Figure 13. EXAFS fits for data collected from FeS_{nano} and mackinawite. (**a**) $\chi(k)*k^3$ of the mackinawite and FeS_{nano} EXAFS experimental data, (**b**) magnitude and (**c**) real part of the Fourier transform of the $\chi(k)*k^3$. Fitting results are listed in supplementary table 11. Good fits for both models were obtained, but the fit for mackinawite only included the Fe-S path. If the Fe-Fe path would have been included in the model, the fit would have yielded a large σ^2 value and the reduced χ^2 would have increased considerably. In contrast, the fit for FeS_{nano} to the model based on the greigite structure allowed the inclusion of the second Fe shell improving the fit and the goodness-of-fit parameters.

Supplementary References

1. Lutz, H. D. & Haeuseler, H. Infrared and Raman spectroscopy in inorganic solids research. *J. of Mol. Struct.* **511–512**, 69-75 (1999).
2. Bourdoiseau, J. A., Jeannin, M., Sabot, R., Rémazeilles, C. & Refait, P. Characterisation of mackinawite by Raman spectroscopy: Effects of crystallisation, drying and oxidation. *Corros. Sci.* **50**, 3247-3255 (2008).
3. Boughriet, A., Figueiredo, R. S., Laureyns, J. & Recourt, P. Identification of newly generated iron phases in recent anoxic sediments: ^{57}Fe Mossbauer and microRaman spectroscopic studies. *J. Chem. Soc., Faraday Trans.* **93**, 3209-3215 (1997).
4. Gaultois, M. W. & Grosvenor, A. P. Coordination-Induced Shifts of Absorption and Binding Energies in the $SrFe_1{-}xZn_xO_3{-}\delta$ System. *J. Phys. Chem. C* **114**, 19822-19829 (2010).
5. Mycroft, J. R., Bancroft, G. M., McIntyre, N. S., Lorimer, J. W. & Hill, I. R. Detection of sulphur and polysulphides on electrochemically oxidized pyrite surfaces by X-ray photoelectron spectroscopy and Raman spectroscopy. *J. Electroanal. Chem. Interfacial Electrochem.* **292**, 139-152 (1990).
6. Bourdoiseau, J. A., Jeannin, M., Rémazeilles, C., Sabot, R. & Refait, P. The transformation of mackinawite into greigite studied by Raman spectroscopy. *J. Raman Spectrosc.* **42** (2011).
7. Mullet, M., Boursiquot, S., Abdelmoula, M., Génin, J.-M. & Ehrhardt, J. J. Surface chemistry and structural properties of mackinawite prepared by reaction of sulfide ions with metallic iron. *Geochim. et Cosmochim. Acta* **66**, 829-836 (2002).
8. Watson, J. H. P. et al. Structural and magnetic studies on heavy-metal-adsorbing iron sulphide nanoparticles produced by sulphate-reducing bacteria. *J. Magn. Magn. Mater.* **214**, 13-30 (2000).
9. Wilke, M., Farges, F., Petit, P. E., Brown, G. E. & Martin, F. Oxidation state and coordination of Fe in minerals: An Fe K-XANES spectroscopic study. *Am. Mineral.***86**, 714-730 (2001).
10. Berry, A. J., O'Neill, H. S. C., Jayasuriya, K. D., Campbell, S. J. & Foran, G. J. XANES calibrations for the oxidation state of iron in a silicate glass. *Am. Mineral.* **88**, 967-977 (2003).
11. Waychunas, G. A., Apted, M. J. & Brown, G. E. X-ray K-edge absorption spectra of Fe minerals and model compounds: Near-edge structure. *Phys. Chem. Min.* **10**, 1-9 (1983).
12. Bajt, S., Sutton, S. R. & Delaney, J. S. X-ray microprobe analysis of iron oxidation states in silicates and oxides using X-ray absorption near edge structure (XANES). *Geochim. et Cosmochim. Acta* **58**, 5209-5214 (1994).
13. Galoisy, L., Calas, G. & Arrio, M. A. High-resolution XANES spectra of iron in minerals and glasses: structural information from the pre-edge region. *Chem. Geol.* **174**, 307-319 (2001).
14. Humphreys, M. C. S. et al. Coupled Interactions between Volatile Activity and Fe Oxidation State during Arc Crustal Processes. *J. of Petrol.* **58**, 795-814 (2015).
15. Prietzel, J., Thieme, J., Eusterhues, K. & Eichert, D. Iron speciation in soils and soil aggregates by synchrotron-based X-ray microspectroscopy (XANES, μ-XANES). *Eur. J Soil Sci.* **58**, 1027-1041(2007).

16. Jeong, H. Y., Lee, J. H. & Hayes, K. F. Characterization of synthetic nanocrystalline mackinawite: crystal structure, particle size, and specific surface area. *Geochim. et Cosmochim. Acta* **72**, 493-505 (2008).
17. Taylor, L.A. & Finger, L.W. Carnegie Institute of Washington Geophysical Laboratory Annual Report **69**, 318–322, (1970).
18. O'Loughlin, E. J., Kelly, S. D., Cook, R. E., Csencsits, R. & Kemner, K. M. Reduction of Uranium(VI) by Mixed Iron(II)/Iron(III) Hydroxide (Green Rust): Formation of UO_2 Nanoparticles. *Environ. Sci. & Technol.* **37**, 721-727 (2003).
19. Hoggins, J. T. & Steinfink, H. Empirical bonding relationships in metal-iron-sulfide compounds. *Inorg. Chem.* **15**, 1682-1685 (1976).
20. McCammon, C. A. Equation of state, bonding character, and phase transition of cubanite, $CuFe_2S_3$, studied from 0 to 5 GPa. *Am. Mineral.* **80**, 1-8 (1995).
21. Shannon, R. D. *16 - Bond Distances in Sulfides and a Preliminary Table of Sulfide Crystal Radii*. In: *Industrial Chemistry Library* (Elsevier, 1981).
22. Gibbs, G. V. et al. Theoretical Electron Density Distributions for Fe- and Cu-Sulfide Earth Materials: A Connection between Bond Length, Bond Critical Point Properties, Local Energy Densities, and Bonded Interactions. *J. Phys. Chem. B* **111**, 1923-1931 (2007).
23. Skinner, B. J., Erd, R. C. & Grimaldi, F. S. Greigite, the thio-spinel of iron; A new mineral. *Am. Mineral.* **49,** 543–555 (1964).
24. Rendon-Diazmiron, L. E. & Steinfink, H. The stabilization of tetravalent iron in tetrahedral sulfur coordination. *J. Solid State Chem.* **27**, 261-262 (1979).
25. Hennig, C. et al. EXAFS as a tool for bond-length determination in the environment of heavy atoms. *J. Synchrotron Rad.* **8**, 695-697 (2001).
26. Terranova, U. & de Leeuw, N. H. A force field for mackinawite surface simulations in an aqueous environment. *Theor. Chem. Acc.* **135**, 46 (2016).
27. Kresse, G. Ab initio molecular dynamics for liquid metals. *J. Non. Cryst. Solids.* **192**, 222-229 (1995).
28. Kresse, G. & Furthmüller, J. Efficiency of ab-initio total energy calculations for metals and semiconductors using a plane-wave basis set. *Comput. Mater. Sci.* **6**, 15-50 (1996).
29. Grimme, S. Semiempirical GGA-type density functional constructed with a long-range dispersion correction. *J. Comput. Chem.* **27**, 1787-1799 (2006).
30. Dzade, N., Roldan, A. & de Leeuw, N. H. The surface chemistry of NO_x on mackinawite (FeS) surfaces: a DFT-D2 study. *Phys. Chem. Chem. Phys.* **16**, 15444-15456 (2014).
31. Perdew, J. P., Burke, K. & Ernzerhof, M. Generalized gradient approximation made simple. *Phys. Rev. Lett.* **77**, 3865-3868 (1996).
32. Blöchl, P. E. Projector augmented-wave method. *Phys. Rev. B* **50**, 17953-17979 (1994).
33. Monkhorst, H. J. & Pack, J. D. Special points for Brillouin-zone integrations. *Phys. Rev. B* **13**, 5188-5192 (1976).
34. Bondi, A. van der Waals Volumes and Radii of Metals in Covalent Compounds. *J. Phys. Chem.* **70**, 3006-3007 (1966).
35. Fujimori, T. *et al.* Conducting linear chains of sulphur inside carbon nanotubes. *Nat. Commun.* **4**, 1-8 (2013).
36. Csákberényi-Malasics, D. et al. Structural properties and transformations of precipitated FeS. *Chem. Geol.* **294–295**, 249-258 (2012).
37. Hagen, M. *et al.* In-situ Raman investigation of polysulfide formation in Li-S cells. *J. Electrochem. Soc.* **160**, A1205-A1214 (2013).

38. El Jaroudi, O., Picquenard, E., Demortier, A., Lelieur, J.P. & Corset, J. Polysulfide Anions. 1. Structure and Vibrational Spectra of the S_2^{2-} and S_3^{2-} Anions. Influence of the Cations on Bond Length and Angle. *Inorg. Chem.* **38**, 2394-2401 (1999).
39. Diao, Y., Xie, K., Xiong, S. & Hong, X. Insights into Li-S battery cathode capacity fading mechanisms: irreversible oxidation of active mass during cycling. *J. Electrochem. Soc.* **159**, A1816-A1821 (2012).
40. Berg, J. S., Schwedt, A., Kreutzmann, A.C., Kuypers, M. M. M. & Milucka, J. Polysulfides as Intermediates in the Oxidation of Sulfide to Sulfate by Beggiatoa spp. *Appl. Environ. Microbiol.* **80**, 629-636 (2014).
41. Pratt, A. R., Muir, I. J. & Nesbitt, H. W. X-ray photoelectron and Auger electron spectroscopic studies of pyrrhotite and mechanism of air oxidation. *Geochim. et Cosmochim. Acta* **58**, 827-841 (1994).
42. van der Heide, H., Hemmel, R., van Bruggen, C. F. & Haas, C. X-ray photoelectron spectra of 3d transition metal pyrites. *J. Solid State Chem.* **33**, 17-25 (1980).
43. Ennaoui, A., Fiechter, S., Jaegermann, W. & Tributsch, H. Photoelectrochemistry of Highly Quantum Efficient Single-Crystalline n-FeS_2 (Pyrite). *J. Electrochem. Soc.* **133**, 97-106 (1986).
44. Buckley, A. N. & Woods, R. X-ray photoelectron spectroscopy of oxidized pyrrhotite surfaces: I. Exposure to aire. *Appl. Surf. Sci.* **22–23,** 280-287 (1985).
45. Jones, C. F., LeCount, S., Smart, R. S. C. & White, T. J. Compositional and structural alteration of pyrrhotite surfaces in solution: XPS and XRD studies. *Appl. Surf. Sci.* **55**, 65-85 (1992).
46. Thomas, J. E., Skinner, W. M. & Smart, R. S. t. C. A comparison of the dissolution behavior of troilite with other iron(II) sulfides; implications of structure. *Geochim. et Cosmochim. Acta* **67**, 831-843 (2003).
47. Herbert Jr, R. B., Benner, S. G., Pratt, A. R. & Blowes, D. W. Surface chemistry and morphology of poorly crystalline iron sulfides precipitated in media containing sulfate-reducing bacteria. *Chem. Geol.* **144**, 87-97 (1998).
48. Boursiquot, S., Mullet, M., Abdelmoula, M., Génin, J. M. & Ehrhardt, J. J. The dry oxidation of tetragonal FeS_{1-x} mackinawite. *Phys. Chem. Min.* **28**, 600-611 (2001).
49. Hyland, M. M. & Bancroft, G. M. An XPS study of gold deposition at low temperatures on sulphide minerals: Reducing agents. *Geochim. et Cosmochim. Acta* **53**, 367-372 (1989).
50. Toniazzo, V., Mustin, C., Vayer-Besancon, M., Erre, R. & Berthelin, J. in *Effect of Mineral-Organic-Microorganism Interactions on Soil and Freshwater Environments*. 37-45 (Springer US, 1999).
51. Buckley, A. N. & Woods, R. The surface oxidation of pyrite. *Appl. Surf. Sci.***27**, 437-452 (1987).
52. Nesbitt, H. W. et al. Synchrotron XPS evidence for Fe^{2+}-S and Fe^{3+}-S surface species on pyrite fracture-surfaces, and their 3D electronic states. *Am. Mineral.* **85**, 850-857 (2000).
53. Smart, R. S. C., Skinner, W. M. & Gerson, A. R. XPS of sulphide mineral surfaces: metal-deficient, polysulphides, defects and elemental sulphur. *Surf. Interface Anal.* **28**, 101-105 (1999).
54. Lennie, A. R. & Vaughan, D. J. Spectroscopic Studies of Iron Sulfide Formation and Phase Relations at Low Temperatures. *Min. Spectros. Special Publication* **5**, 117-131 (1996).

55. Buckley, A. N., Woods, R. & Wouterlood, H. J. An XPS investigation of the surface of natural sphalerites under flotation-related conditions. *Int. J. Miner. Process.* **26**, 29-49 (1989).
56. Shalvoy, R. B., Fisher, G. B. & Stiles, P. J. Bond ionicity and structural stability of some average-valence-five materials studied by x-ray photoemission. *Phys. Rev. B* **15**, 1680-1697 (1977).
57. Hansson, E. B., Odziemkowski, M. & Gillham, R. Formation of poorly crystalline iron monosulfides: surface redox reactions on high purity iron, spectroelectrochemical studies. *Corros. Sci.* **48**, 3767-3783 (2006).
58. Boursiquot, S., Mullet, M. & Ehrhardt, J.J. XPS study of the reaction of chromium (VI) with mackinawite (FeS). *Surf. Interface Anal.* **34**, 293-297 (2002).
59. Sabri, M. et al. Hydroxyl radical-assisted decomposition and oxidation in solution-processed indium oxide thin-film transistors. *J. Mat. Chem. C* **3**, 7499-7505 (2015).
